# Interaction of the hydrogen molecule with the environment: stability of the system and the ${\mathscr{P}}{\mathscr{T}}$ symmetry breaking

**DOI:** 10.1038/s41598-019-56849-2

**Published:** 2020-01-14

**Authors:** I. A. Wrona, M. W. Jarosik, R. Szczȩśniak, K. A. Szewczyk, M. K. Stala, W. Leoński

**Affiliations:** 1 Institute of Physics, Jan Długosz University in Czȩstochowa, Ave. Armii Krajowej 13/15, 42-200 Czȩstochowa, Poland; 2 Institute of Physics, Jan Długosz University, Ave. Armii Krajowej 19, 42-200 Czȩstochowa, Poland; 3 Quantum Optics and Engineering Division, Faculty of Physics and Astronomy, University of Zielona Góra, Prof. Z. Szafrana 4a, 65-516 Zielona Góra, Poland; 4 Joint Laboratory of Optics of Palacký University and Institute of Physics of CAS, RCPTM, Faculty of Science, Palacký University, 17. listopadu 12, 771 46 Olomouc, Czech Republic

**Keywords:** Physics, Theoretical physics

## Abstract

The stability of the hydrogen molecule interacting with the environment according to the balanced gain and loss energy scheme was studied. We determined the properties of the molecule taking into account all electronic interactions, the parameters of the Hamiltonian being computed by the variational method. The interaction of the hydrogen molecule with the environment was modeled parametrically (*γ*) by means of the non-Hermitian, ${\mathscr{P}}{\mathscr{T}}$-symmetric Hamiltonian. We showed that the hydrogen molecule is dynamically unstable. Its dissociation time (*T*_*D*_) decreases if the *γ* parameter increases (for *γ* → 0 we got *T*_*D*_ → + ∞). The dynamic instability of the hydrogen molecule is superimposed on the decrease in its static stability as *γ* increases. Then we can observe the decrease in the dissociation energy value and the existence of the metastable state of the molecule as *γ*_*MS*_ reaches 0.659374 Ry. The hydrogen molecule is statically unstable when *γ* > *γ*_*D*_ = 1.024638 Ry. Moreover, we can also observe the ${\mathscr{P}}{\mathscr{T}}$ symmetry breaking effect for the electronic Hamiltonian when ${\gamma }_{{\mathscr{P}}{\mathscr{T}}}$ = 0.520873 Ry. This effect does not affect such properties of the hydrogen molecule as: the electronic Hamiltonian parameters, the phonon and the rotational energies, and the values of the electron-phonon coupling constants neither it disturbs the dynamics of the electronic subsystem. However, the number of available quantum states goes down to four.

## Introduction

Research on the impact of the environment (external quantum system) on the state of the quantum system is an interesting, but very difficult issue^1,2^. This is due to two reasons: (i) usually in the case of the realistic quantum system it is impossible to accurately determine its internal state due to the mathematical complexity of the problem, and (ii) the interaction between the quantum system and the environment can be so complicated that it is impossible to obtain unambiguous results.

In the paper, we studied the physical properties of the hydrogen molecule, which interacts with the environment according to the Balanced Gain and Loss (BGL) energy scheme^3^. The hydrogen molecule is an interesting case because it represents the non-trivial quantum system, and its state can be described accurately using the variational method^4–7^. On the other hand, the BGL scheme describes the interaction between the molecule and the environment in the realistic and simple way. From the mathematical point of view, the BGL type interaction is modeled by the non-Hermitian Hamiltonian^8,9^ which is invariant due to the ${\mathscr{P}}{\mathscr{T}}$ symmetry (the symmetry of reflection in space (${\mathscr{P}}$) and in time (${\mathscr{T}}$))^10–14^. It should be emphasized that if the Hamiltonian is the non-Hermitian one, but has the unbroken ${\mathscr{P}}{\mathscr{T}}$ symmetry, the energy spectrum of the system is real – at least to the characteristic value of the parameter controlling the interaction with the environment.

The interest in the non-Hermitian Hamiltonians, in the context of the description of the open systems, appeared in many areas of physics. The papers^15,16^, in which the open Bose-Hubbard dimer was analyzed are worth mentioning here. Such system can be implemented experimentally in the form of trapped bosons, where the coupling constant between the studied system and the environment reflects the value of barrier potential^17^. The ${\mathscr{P}}{\mathscr{T}}$ symmetry breaking was also analyzed in^18–20^ in the context of Bose-Einstein condensates. It is worth noting that the existence of the ${\mathscr{P}}{\mathscr{T}}$ symmetry breaking effects were also confirmed in the field of quantum optics^21–24^. Additionally, the fact of the appearance of complex energy values can be applied in explanation of the dynamics of the physical systems, the probability of disintegration, or the transport mechanism^25–31^. It should be noticed that although most of the existing models were introduced heuristically, nevertheless it was done on the basis of relatively satisfactory mathematical justification^17^.

On the basis of the discussed issues, we intended to analyze the hydrogen molecule interacting with the environment and to examine its stability. We assumed that the required calculations would be carried out in the extremely accurate manner (at the level required by the the quantum chemistry standards), so that the obtained results could be verified experimentally.

In our opinion, the results presented in the paper shall be useful for people interested in the statistical and dynamical stability of small quantum systems interacting with environment and for those who research into the impact of ${\mathscr{P}}{\mathscr{T}}$ symmetry breaking on the physical properties of a system. The presented results are significant insomuch that they concern the real physical system, and calculations were carried out for the model which does not contain any free parameters.

The achieved results can also be important from the technical point of view, because they can be related to the properties of the electronic devices in the single-molecule scale. Indeed, we already know that single particles can act similarly to the crucial elements of contemporary microelectronics, in particular they can serve as the rectifiers^32^, the electronic mixers^33^, and the switchers^34–36^. Therefore one can reasonably hope that the molecular electronics will replace the current technologies over time. However, the real development in this branch of knowledge depend on the full understanding of the transport mechanisms in single-molecule junctions. The presented work, by the example of the hydrogen molecule interacting with the environment in the balanced gain and loss energy scheme, draws attention to the fact that the interaction of the molecular bridge with anchors of the nanojunction can lead to changes in the bridge energy levels and to the reduction of their number. This is a substantial effect, because the electronic structure of a single molecule controls the electrical properties of the junction, in which it is used as a building block^37^. The way of description of physical properties of the molecular bridge in the nanojunction, applied within the formalism presented in the work, is discussed in detail in the concluding part of the work.

## Formalism

The total energy (*E*_*T*_) of hydrogen molecule is defined as:

1 $${E}_{T}={E}_{p}+{E}_{e\gamma },$$

where: *E*_*p*_ = 2/*R* represents the energy of proton repulsion, with *R* = |**R**| as the distance between protons, and *E*_*eγ*_ means the energy of the lowest electronic state in the presence of the loss and gain effect (*γ* represents the coupling between the molecule and the environment). For *γ* = 0, the energy ${E}_{e}={E}_{e(\gamma \mathrm{=0})}$ should be determined using the Hubbard Hamiltonian, which takes into account all electronic interactions. In the second quantization formalism, we have^6^:

2 $$\begin{array}{rcl}{\hat{\mathscr{H}}}_{e} & = & \varepsilon ({\hat{n}}_{1}+{\hat{n}}_{2})+t\sum _{\sigma }\,({\hat{c}}_{1\sigma }^{\dagger }{\hat{c}}_{2\sigma }+{\hat{c}}_{2\sigma }^{\dagger }{\hat{c}}_{1\sigma })+U({\hat{n}}_{1\uparrow }{\hat{n}}_{1\downarrow }+{\hat{n}}_{2\uparrow }{\hat{n}}_{2\downarrow })\\  &  & +\,(K-\frac{J}{2}){\hat{n}}_{1}{\hat{n}}_{2}-2J{\hat{{\bf{S}}}}_{1}{\hat{{\bf{S}}}}_{2}+J({\hat{c}}_{1\uparrow }^{\dagger }{\hat{c}}_{1\downarrow }^{\dagger }{\hat{c}}_{2\downarrow }{\hat{c}}_{2\uparrow }+h.\,c\mathrm{}.)\\  &  & +\,V\sum _{\sigma }\,[({\hat{n}}_{1-\sigma }+{\hat{n}}_{2-\sigma })({\hat{c}}_{1\sigma }^{\dagger }{\hat{c}}_{2\sigma }+{\hat{c}}_{2\sigma }^{\dagger }{\hat{c}}_{1\sigma })],\end{array}$$

where ${\hat{n}}_{j}$ is given by: ${\hat{n}}_{j}={\sum }_{\sigma }{\hat{n}}_{j\sigma }={\sum }_{\sigma }{\hat{c}}_{j\sigma }^{\dagger }{\hat{c}}_{j\sigma }$, and ${\hat{c}}_{j\sigma }^{\dagger }$ (${\hat{c}}_{j\sigma }$) is the electron creation (annihilation) operator, which refers to the *j*-th hydrogen atom, *σ* represents the electronic spin: $\sigma \in \{\uparrow ,\downarrow \}$. In the last part of the Hamiltonian, ${\hat{\mathscr{H}}}_{e}$ the symbol −*σ* (in the subscript) denotes the spin direction opposite to the direction marked with *σ*. The product of spin operators ${\hat{{\bf{S}}}}_{i}{\hat{{\bf{S}}}}_{j}$ takes the form of: $\frac{1}{2}({\hat{S}}_{i}^{+}{\hat{S}}_{j}^{-}+{\hat{S}}_{i}^{-}{\hat{S}}_{j}^{+})+{\hat{S}}_{i}^{z}{\hat{S}}_{j}^{z}$, where ${\hat{S}}_{j}^{+}={\hat{c}}_{j\uparrow }^{\dagger }{\hat{c}}_{j\downarrow }$, ${\hat{S}}_{j}^{-}={\hat{c}}_{j\downarrow }^{\dagger }{\hat{c}}_{j\uparrow }$, and ${\hat{S}}_{j}^{z}=\frac{1}{2}({\hat{n}}_{j\uparrow }-{\hat{n}}_{j\downarrow })$. The symbol +*h*.*c*. in Eq. (2) is the abbreviation for *plus the Hermitian conjugate* and it means that an additional term being the Hermitian conjugate of the preceding term should be added. The Hamiltonian parameters are defined by the following integrals:

3 $$\begin{array}{rcl}\varepsilon  & = & \int \,{d}^{3}{\bf{r}}{\Phi }_{1}({\bf{r}})[-{\nabla }^{2}-\frac{2}{|{\bf{r}}-{\bf{R}}|}]{\Phi }_{1}({\bf{r}}),\\ t & = & \int \,{d}^{3}{\bf{r}}{\Phi }_{1}({\bf{r}})[-{\nabla }^{2}-\frac{2}{|{\bf{r}}-{\bf{R}}|}]{\Phi }_{2}({\bf{r}}),\\ U & = & \int \int \,{d}^{3}{{\bf{r}}}_{1}{d}^{3}{{\bf{r}}}_{2}{\Phi }_{1}^{2}({{\bf{r}}}_{1})\frac{2}{|{{\bf{r}}}_{1}-{{\bf{r}}}_{2}|}{\Phi }_{1}^{2}({{\bf{r}}}_{2}),\\ K & = & \int \int \,{d}^{3}{{\bf{r}}}_{1}{d}^{3}{{\bf{r}}}_{2}{\Phi }_{1}^{2}({{\bf{r}}}_{1})\frac{2}{|{{\bf{r}}}_{1}-{{\bf{r}}}_{2}|}{\Phi }_{2}^{2}({{\bf{r}}}_{2}),\\ J & = & \int \int \,{d}^{3}{{\bf{r}}}_{1}{d}^{3}{{\bf{r}}}_{2}{\Phi }_{1}({{\bf{r}}}_{1}){\Phi }_{2}({{\bf{r}}}_{1})\frac{2}{|{{\bf{r}}}_{1}-{{\bf{r}}}_{2}|}{\Phi }_{1}({{\bf{r}}}_{2}){\Phi }_{2}({{\bf{r}}}_{2}),\\ V & = & \int \int \,{d}^{3}{{\bf{r}}}_{1}{d}^{3}{{\bf{r}}}_{2}{\Phi }_{1}^{2}({{\bf{r}}}_{1})\frac{2}{|{{\bf{r}}}_{1}-{{\bf{r}}}_{2}|}{\Phi }_{1}({{\bf{r}}}_{1}){\Phi }_{2}({{\bf{r}}}_{2})\mathrm{}.\end{array}$$

The meaning of above quantities is as follows: *ε* represents the energy of the molecular orbital, *t* is the electronic hopping integral, *U* denotes the on-site Coulomb repulsion, *K* is the energy of the inter-site Coulomb repulsion, *J* stands for the integral of the exchange, and *V* is called the correlated hopping. The integrals were calculated numerically, which is the complicated procedure that requires the use of the large computer resources. We notice that the contribution of the individual integrals to the energy eigenvalues is very diverse (see Table 1), nevertheless omitting any interaction would lead to the non-physical shortening of the distance between protons. We chose the Wannier’s functions in the form of:

4 $${\Phi }_{j}(r)=a[{\phi }_{j}({\bf{r}})-b{\phi }_{l}({\bf{r}})],$$

where the coefficients ensuring normalization are expressed in the formulas:

5 $$a=\frac{1}{\sqrt{2}}\sqrt{\frac{1+\sqrt{1-{S}^{2}}}{1-{S}^{2}}},\,b=\frac{S}{1+\sqrt{1-{S}^{2}}}\mathrm{}.$$

**Table 1:** The values of the Hubbard Hamiltonian integrals calculated for the equilibrium distance of the hydrogen molecule. The selected values *γ* have been taken into account.

*γ* [Ry]	*ε*_0_ [Ry]	*t*_0_ [Ry]	*U*_0_ [Ry]	*K*_0_ [Ry]	*J*_0_ [Ry]	*V*_0_ [Ry]
**0**	**−1.749493**	**−0.737679**	**1.661254**	**0.962045**	**0.022040**	**−0.011851**
0.1	**−**1.74866	**−**0.743562	1.66607	0.965198	0.022117	**−**0.0118825
0.2	**−**1.74599	**−**0.760758	1.68006	0.974349	0.0223398	**−**0.0119744
0.3	**−**1.74114	**−**0.788025	1.70198	0.988664	0.0226876	−0.0121193
0.4	**−**1.73366	**−**0.823632	1.73016	1.00702	0.0231322	**−**0.0123074
0.5	**−**1.72329	**−**0.865608	1.76279	1.02821	0.0236434	**−**0.0125275
${\gamma }_{{\mathscr{P}}{\mathscr{T}}}$ **= 0.520873**	**−1.72075**	**−0.874986**	**1.770000**	**1.03288**	**0.0237558**	**−0.0125764**
0.6	**−**1.70997	**−**0.911931	1.79817	1.05107	0.0241927	**−**0.0127687
*γ*_*MS*_ = **0.659374**	**−1.70076**	**−0.940627**	**1.81984**	**1.064996**	**0.0245259**	**−0.0129176**
0.7	**−**1.69397	**−**0.960458	1.83473	1.07453	0.0247533	**−**0.0130205
0.8	**−**1.67604	**−**1.00854	1.87081	1.09738	0.0252963	**−**0.0132714
0.9	**−**1.65787	**−**1.0515	1.90394	1.11776	0.0257748	**−**0.0135041
0.0	**−**1.6462	**−**1.07108	1.92509	1.1285	0.0260069	**−**0.0136584
*γ*_*D*_ = **1.024638**	**−1.65569**	**−1.03796**	**1.911296**	**1.11581**	**0.0256683**	**−0.0135781**

The atomic overlap (*S*) has the form: $S=\int \,{d}^{3}r{\phi }_{1}({\bf{r}}){\phi }_{2}({\bf{r}})$, where 1 *s* Slater-type orbital can be written as: ${\phi }_{j}({\bf{r}})=\sqrt{{\alpha }^{3}/\pi }\,\exp [-\,\alpha |{\bf{r}}-{{\bf{R}}}_{j}|]$, *α* is the inverse size of the orbital. It should be noted that the second quantization method is completely equivalent to the Schrödinger analysis^38–42^.

The effective interaction of hydrogen molecule with the environment will be taken into account by supplementing the Hubbard Hamiltonian ${\hat{\mathscr{H}}}_{e}$ with the balanced gain and loss operator^3,43^:

6 $${\hat{\mathscr{H}}}_{\gamma }=i\gamma ({\hat{n}}_{1}-{\hat{n}}_{2})\mathrm{}.$$

The interpretation is that the operators $i\gamma {\hat{n}}_{1}$ and $-i\gamma {\hat{n}}_{2}$ enable the effective description of the inward and outward fluxes of the probability amplitude (the interaction with the environment)^3,44^. The addition or removal of an electron from the system would look more realistic, but this approach requires solving the master equations^45–47^, which is a quite complicated numerical task. Notice that the operator ${\hat{\mathscr{H}}}_{e\gamma }={\hat{\mathscr{H}}}_{e}+{\hat{\mathscr{H}}}_{\gamma }$ represents the non-Hermitian Hamiltonian, nevertheless it remains invariant due to the ${\mathscr{P}}{\mathscr{T}}$ symmetry – at least to the characteristic ${\gamma }_{{\mathscr{P}}{\mathscr{T}}}$ value for which the symmetry is broken.

## Results

### Static stability of the system: the electron, the phonon and the electron-phonon properties

**Figure 1:**
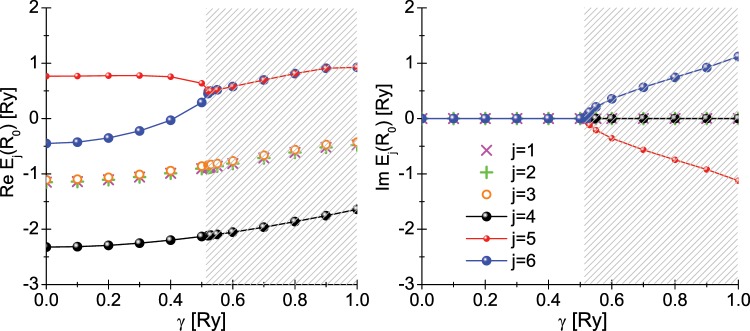
The real and the imaginary part of the eigenvalues of the Hamiltonian ${\hat{\mathscr{H}}}_{e\gamma }$. The equilibrium distances between protons (*R*_0_) were assumed. The hatched areas correspond to the $\gamma$ values for which the operator ${\hat{\mathscr{H}}}_{e\gamma }$ ceases to be invariant due to the ${\mathscr{P}}{\mathscr{T}}$ symmetry.

In the Fig. 1, we plotted the dependence of the eigenvalues *E*_*j*_ on *γ*. The analytical formulas for *E*_*j*_ have been collected in the Appendix. Analyzing the obtained results, we found that for ${\gamma }_{{\mathscr{P}}{\mathscr{T}}}=0.520873$ Ry there occurs the breaking of ${\mathscr{P}}{\mathscr{T}}$ symmetry of the electronic Hamiltonian. This fact is manifested by the appearance of the complex values of *E*_5_ and *E*_6_. Physically, this means that the ${\mathscr{P}}{\mathscr{T}}$ symmetry breaking reduces the number of the available electronic states from six to four. Nevertheless, the considered effect has no physical significance due to the fact that the states $|{E}_{5}\rangle$ and $|{E}_{6}\rangle$ have the highest energy values. They cannot be thermally occupied - the *k*_*B*_*T* energy is of the order of 25 meV, while the difference between *E*_6_ and *E*_4_ is around 25.5 eV (*E*_4_ is the ground state energy of the electronic subsystem). When discussing the results, it should be clearly emphasized that *E*_4_ is always the real number.

**Figure 2:**
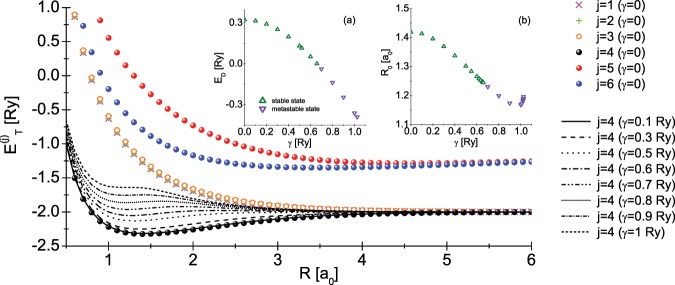
The dependence of the total energy ${E}_{T}^{(j)}$ on the distance between protons. Additionaly, we took into account the influence of *γ* on the ground state energy ${E}_{T}^{(4)}$. Insert (**a**) the dissociation energy *E*_*D*_ versus *γ* parameter. Insert (**b**) equilibrium distance *R*_0_ versus *γ* parameter.

**Figure 3:**
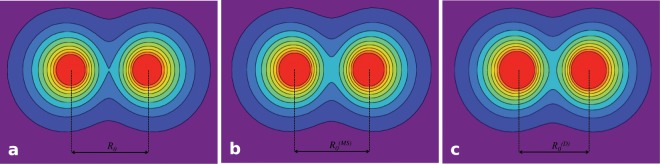
The distribution of electronic charge in the hydrogen molecule: (**a**) the stable case; (**b**) metastable case; (**c**) just before dissociation.

Although the ${\mathscr{P}}{\mathscr{T}}$ symmetry breaking is not manifested physically, the interaction of the hydrogen molecule with the environment can significantly change its physical state. This fact is connected with the dependence of the total energy on the *γ* parameter. In the Fig. 2, we presented the total energy values (${E}_{T}^{(j)}={E}_{p}+{E}_{j}$) of the isolated hydrogen molecule, and the influence of the *γ* parameter on the ground state energy ${E}_{T}^{(4)}$. One can see that an increase in the minimum energy value ${E}_{T}^{(4)}({R}_{0})$ is observed with the increase in *γ*, whereas the molecule is in the stable state. Above ${\gamma }_{MS}=0.659374$ Ry the hydrogen molecule can exist only in the metastable state: ${E}_{T}^{(4)}({R}_{0}^{(MS)}) > {E}_{T}^{(4)}(R\to +\infty )=2$ Ry, where ${R}_{0}^{(MS)}=1.244701$ a_0_. After exceeding *γ*_*D*_ = 1.024638 Ry, which corresponds to ${R}_{0}^{(D)}=1.196587$ a_0_, the molecule breaks down (see the appendix). The insert (a) presents the dependence of the hydrogen dissociation energy (${E}_{{\rm{D}}}=2{\rm{R}}y-{E}_{T}^{(4)}$) on the value of the *γ* parameter. The insert (b) shows the influence of the *γ* parameter on the equilibrium distance *R*_0_. Figure 3(a–c) trace the change of the distribution of electron charge for the stable case at *γ* = 0 (*R*0 = 1.41968 a_0_), for the metastable case (${R}_{0}^{(MS)}$), and at the dissociation point (${R}_{0}^{(D)}$). The density of electron charge was calculated according to the formula: $\rho ({\bf{r}})={\sum }_{j}|{\Phi }_{j}({\bf{r}}){|}^{2}$.

The determination of the explicit function *E*_*T*_(*R*) for the given parameter *γ* allows to trace the influence of the environment on the vibrational energy. In the simplest approach (the harmonic approximation), the potential can be calculated as follows: ${V}_{{\rm{H}}}(R)={E}_{T}^{(4)}({R}_{0})+\frac{1}{2}{k}_{{\rm{H}}}{(R-{R}_{0})}^{2}$, where: ${k}_{{\rm{H}}}={[{d}^{2}{E}_{T}^{(4)}(R)/d{R}^{2}]}_{R={R}_{0}}$. The energy of quantum oscillator has the form:

7 $${E}_{o}^{{\rm{H}}}={\omega }_{0}^{{\rm{H}}}(n+\mathrm{1/2})\mathrm{}.$$

The symbol *n* indexes the energy level: $n=\mathrm{0,}\,\mathrm{1,}\,\mathrm{2,}\,\mathrm{..}.$. Additionally, ${\omega }_{0}^{{\rm{H}}}=\sqrt{{k}_{{\rm{H}}}/m^{\prime} }$, where *m*′ is the reduced mass of the protons: $m^{\prime} ={m}_{p}/2=918.076336$ (*m*_*p*_ is the proton mass). The more advanced approach is based on the Morse potential: ${V}_{{\rm{M}}o}(R)={E}_{T}^{(4)}({R}_{0})+{E}_{{\rm{D}}}{[1-\exp (-{\alpha }_{{\rm{M}}o}(R-{R}_{0}))]}^{2}$, where *α*_Mo_ means measure of the curvature of the potential about its minimum. The force constants, *k*_Mo_ should be calculated according to the formula: ${k}_{{\rm{Mo}}}={[{d}^{2}{V}_{{\rm{Mo}}}(R)/d{R}^{2}]}_{R={R}_{0}}$. The Morse energy is given by: ${\omega }_{0}^{{\rm{Mo}}}=\sqrt{{k}_{{\rm{M}}o}/m^{\prime} }$ (see Table 2). The energy formula has the more complex form than for the harmonic case:

8 $${E}_{o}^{{\rm{Mo}}}={\omega }_{0}^{{\rm{Mo}}}(n+\mathrm{1/2})+({({\omega }_{0}^{{\rm{Mo}}})}^{2}\mathrm{/4}{E}_{D}){(n+\mathrm{1/2})}^{2}\mathrm{}.$$

**Table 2:** The Morse potential parameters for different values of *γ*.

*γ* [Ry]	*E*_D_ [Ry]	*α*_M_ [${{\bf{a}}}_{{\bf{0}}}^{-{\bf{1}}}$]
**0**	**0.323007**	**1.441564**
0.1	0.314916	1.19386
0.2	0.290867	1.24564
0.3	0.251537	1.33815
0.4	0.19483	1.48597
0.5	0.123658	1.76235
${\gamma }_{{\mathscr{P}}{\mathscr{T}}}$ = **0.520873**	**0.114146**	**1.816998**
0.6	0.062886	2.19766
${\gamma }_{{\mathcal M}{\mathcal S}}$ = **0.659374**	**0.0194576**	**2.95769**

**Figure 4:**
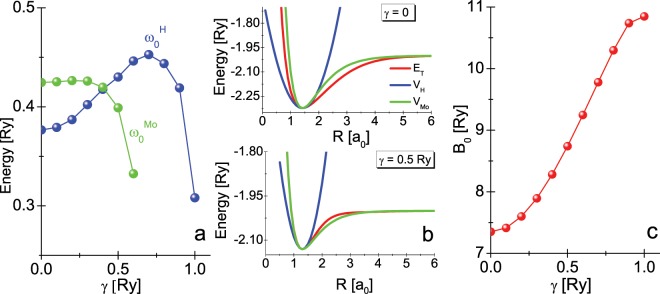
(**a**) The influence of the *γ* parameter on the energy values ${\omega }_{0}^{{\rm{H}}}$ and ${\omega }_{0}^{{\rm{Mo}}}$. (**b**) The exemplary parameterization of the total energy curve in the harmonic and the anharmonic Morse case. (**c**) The rotational constant *B*_0_ versus *γ* parameter.

Figure 4(a) depicts the dependence of the energies ${\omega }_{0}^{{\rm{H}}}$ and ${\omega }_{0}^{{\rm{Mo}}}$ on the value of the *γ* parameter. There is a clear difference in the courses of the functions under consideration. It results from the applied method of approximation of the exact dependence of the total energy on the inter-proton distance (see Fig. 4(b)). It is worth noticing that the anharmonic approximation can be used only for the *γ* values smaller than *γ*_*MS*_; the Morse curve incorrectly parameterizes the ground state energy function ${E}_{T}^{(4)}(R)$ for higher values of *γ*.

The rotational energy of the hydrogen molecule should be calculated from the expression:

9 $${E}_{r}={B}_{0}l(l+1),$$

where: ${B}_{0}=\mathrm{1/}m^{\prime} {R}_{0}^{2}$ and $l=0,\,1,\,2,\,\mathrm{..}.$. The influence of the *γ* parameter on the rotational energy value has been presented in the Fig. 4(c). From the physical point of view, the increase of the energy *B*_0_ results from the decrease of the equilibrium distance *R*_0_, which we observe when the *γ* parameter grows (see the Fig. 2 - insert (b)).

**Figure 5:**
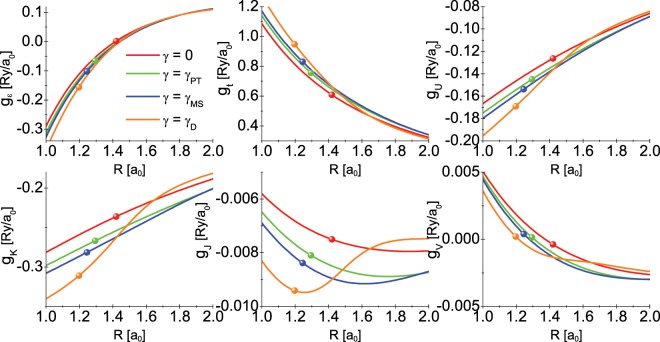
The electron-phonon coupling as a function of inter-proton distance for selected values of the *γ* parameter. The symbols placed on the curves point to the equilibrium value of the inter-proton distance.

Having the explicit dependence of the ${\hat{\mathscr{H}}}_{e\gamma }$ parameters on *R* (see the appendix), we computed the electron-phonon coupling functions according to the formula: *g*_*x*_ = *dx*/*dR*, where $x\in \{\varepsilon ,t,U,K,J,V\}$. We plotted the obtained results in the Fig. 5. One can easily see that the absolute values of the considered functions at *R*_0_ increase as the *γ* parameter increases. The couplings associated with the *ε*, *t*, *U*, and *K* parameters are of the greatest physical importance. The other two quantities *g*_*J*_ and *g*_*V*_ take very small values as compared to other electron-phonon coupling functions. Note the relatively high values of the *g*_*U*_ and *g*_*K*_ functions. The obtained result is caused by the fact that the electrons in the hydrogen molecule form the strongly correlated system.

### The dynamic instability of the hydrogen molecule

The basic observable of the electronic subsystem is the occupation number $\langle {\hat{n}}_{j\sigma }\rangle$ of the *j*^*th*^ proton of the hydrogen molecule, where the symbol $\langle \mathrm{..}.\rangle$ means the expectation value. In the Hermitian case (*γ* = 0) the dynamics of $\langle {\hat{n}}_{j\sigma }\rangle$ can be analyzed using the conventional Heisenberg equation:

10 $$i\frac{d\langle {\hat{n}}_{j\sigma }\rangle }{dT}=\langle {[{\hat{n}}_{j\sigma },{\hat{\mathscr{H}}}_{e\gamma }^{MF}]}_{-}\rangle ,$$

where ${\hat{\mathscr{H}}}_{e\gamma }^{MF}$ is the electron Hamiltonian in the mean-field approximation:

11 $${\hat{\mathscr{H}}}_{e\gamma }^{MF}=\sum _{j\sigma }\,{\varepsilon }_{j\sigma }(\gamma ){\hat{n}}_{j\sigma }+\sum _{j\sigma }\,{t}_{j\sigma }{\hat{n}}_{j\overline{j}\sigma }+\sum _{j\sigma }\,{J}_{j\sigma }{\hat{n}}_{j\sigma -\sigma }+\sum _{j}({P}_{j}{\hat{\Delta }}_{j}^{\dagger }+{P}_{j}^{\ast}{\hat{\Delta }}_{j})\mathrm{}.$$

The Hamiltonian parameters were defined by the expressions:

12 $$\begin{array}{rcl}{\varepsilon }_{j\sigma }(\gamma ) & = & \varepsilon +U\langle {\hat{n}}_{j-\sigma }\rangle +K\sum _{\sigma ^{\prime} }\,\langle {\hat{n}}_{\overline{j}\sigma ^{\prime} }\rangle -J\langle {\hat{n}}_{\overline{j}\sigma }\rangle \\  &  & +\,V[\langle {\hat{n}}_{j\overline{j}-\sigma }\rangle +\langle {\hat{n}}_{\overline{j}j-\sigma }\rangle ]-{(-1)}^{j}i\gamma ,\\ {t}_{j\sigma } & = & t+V[\langle {\hat{n}}_{j-\sigma }\rangle +\langle {\hat{n}}_{\overline{j}-\sigma }\rangle ],\\ {J}_{j\sigma } & = & -J\langle {\hat{n}}_{\overline{j}-\sigma \sigma }\rangle ,\\ {P}_{j} & = & J\langle {\hat{\Delta }}_{\overline{j}}\rangle \mathrm{}.\end{array}$$

The new symbols have the following meanings:

13 $$\bar{j}=\{\begin{array}{lll}1 & {\rm{for}} & j=2\\ 2 & {\rm{for}} & j=\mathrm{1,}\end{array}$$

$${\hat{n}}_{j\overline{j}\sigma }={\hat{c}}_{j\sigma }^{\dagger }{\hat{c}}_{\overline{j}\sigma }$$, $${\hat{n}}_{j\sigma -\sigma }={\hat{c}}_{j\sigma }^{\dagger }{\hat{c}}_{j-\sigma }$$, and $${\hat{\Delta }}_{j}={\hat{c}}_{j\downarrow }{\hat{c}}_{j\uparrow }$$.

It should be emphasized that for ${\hat{\mathscr{H}}}_{e\gamma }$ the required operator calculations are not feasible due to their extensiveness. The mean-field approximation transforms the operator ${\hat{\mathscr{H}}}_{e\gamma }$ into the Hamiltonian, in which the energy of the molecular state and the hopping integral explicitly depend on the proton index *j* and the spin *σ*. In addition, the Hamiltonian have the part that models the reversal of the spin due to the exchange interaction *J*. It is also worth paying attention to the quantity of ${\hat{\Delta }}_{j}$, which has the formal structure of the Cooper pair annihilation operator in the real space. This analogy is not complete, because the Hamiltonian ${\hat{\mathscr{H}}}_{e\gamma }^{MF}$ term containing ${\hat{\Delta }}_{j}$ and ${\hat{\Delta }}_{j}^{\dagger }$ does not correspond to BCS pairing operator^48–50^ (the integral of the exchange *J*_0_ has the positive value instead of negative - see Table 1).

After performing the required operator calculations, we get the set of sixteen first-order differential equations, which is explicitly written in the appendix. In the non-Hermitian case (*γ* ≠ 0), determining the time dependence of the electron observables is the more subtle issue^16,25,51^. First of all, one must define the operators: ${\hat{\mathscr{H}}}_{e\gamma \pm }^{MF}=\frac{1}{2}({\hat{\mathscr{H}}}_{e\gamma }^{MF}\pm {\hat{\mathscr{H}}}_{e\gamma }^{MF\dagger })$, where ${\hat{\mathscr{H}}}_{e\gamma \pm }^{MF}=\pm \,{\hat{\mathscr{H}}}_{e\gamma \pm }^{MF\dagger }$. Then we use the generalized form of the Heisenberg equation:

14 $$i\frac{d\langle {\hat{n}}_{j\sigma }\rangle }{dT}=\langle {[{\hat{n}}_{j\sigma },{\hat{\mathscr{H}}}_{e\gamma +}^{MF}]}_{-}\rangle +\langle {[{\hat{n}}_{j\sigma },{\hat{\mathscr{H}}}_{e\gamma -}^{MF}]}_{+}\rangle -2\langle {\hat{n}}_{j\sigma }\rangle \langle {\hat{\mathscr{H}}}_{e\gamma -}^{MF}\rangle \mathrm{}.$$

Tedious, but not difficult operator calculations lead to the complex system of the differential equations, which is presented in the appendix in the explicit form.

**Figure 6:**
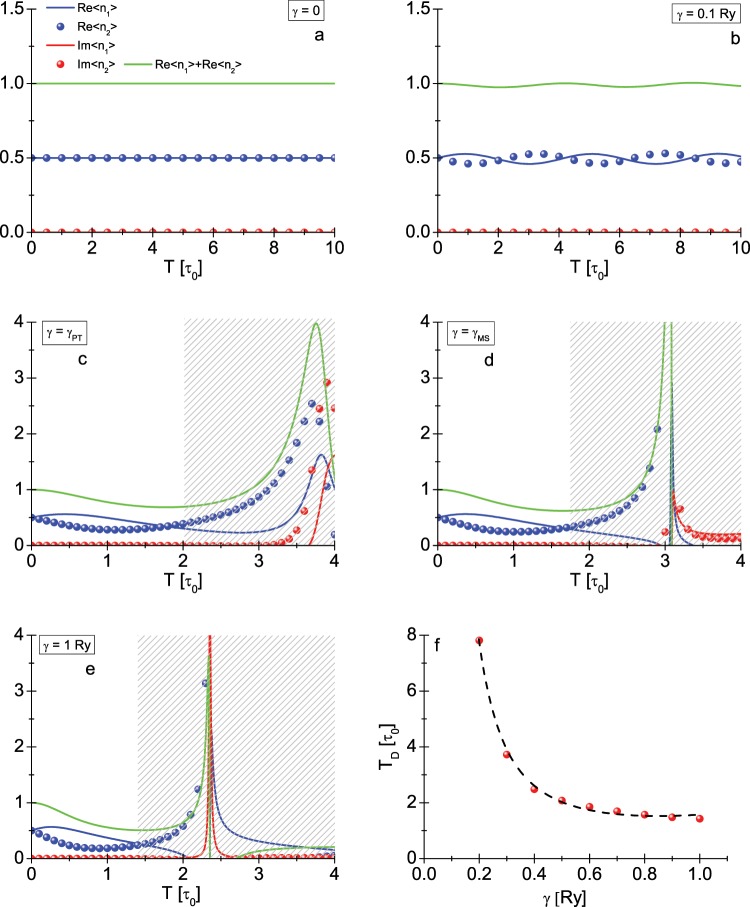
(**a–e**) The time evolution of $\langle {n}_{1\uparrow }\rangle$ and $\langle {n}_{2\uparrow }\rangle$, respectively, for *γ* equal to: 0, 0.1 Ry, ${\gamma }_{{\mathscr{P}}{\mathscr T}}$, ${\gamma }_{{\mathcal M}{\mathcal S}}$, and 1 Ry. The hatched areas correspond to the value greater than *T*_*D*_. *T*_*D*_ is a time, after which an imaginary part of the observables exceeds the value of ±0.005. Figure (**f**) shows the form of *T*_*D*_(*γ*) function. The dashed curve was obtained from the formula: ${T}_{D}=\frac{a}{{\gamma }^{b}}\exp (c\gamma )$, where *a* = 0.095689, *b* = 2.38294, and *c* = 2.80013.

We plotted the time dependence of the observables $\langle {n}_{1\uparrow }\rangle$, $\langle {n}_{2\uparrow }\rangle$ for the selected values of the *γ* parameter in the Fig. 6(a–e). As expected, the system in the Hermitian case is in the stable state, which manifests itself by the time invariance of the expectation value. In the non-Hermitian case, the weak interaction of the hydrogen molecule with the environment (*γ* = 0.1) causes oscillatory changes of the discussed quantities in time. However, this is not time-stable state of the system, because from the specific moment *T*_*D*_ observables $\langle {n}_{1\uparrow }\rangle$, $\langle {n}_{2\uparrow }\rangle$ accept complex values. From the physical point of view, the time *T*_*D*_ should be interpreted as the moment in which the system dissociates. It is easy to show that as the *γ* parameter increases, the oscillations of the expectation values disappear and the value *T*_*D*_ decreases very clearly (see Fig. 6(f)). The obtained results mean that any weak interaction of the hydrogen molecule with the environment modeled in the BGL scheme leads to the finite life time of the molecule.

## Summary and Discussion of the Results

The obtained results show that the BGL type interaction of the hydrogen molecule with the environment leads to its dissociation. From the physical point of view, this means that the hydrogen molecule breaks down into two hydrogen atoms. Note that if the interaction of the hydrogen molecule with the environment would be modelled in the unbalanced gain and loss energy scheme, two other final states could be obtained: ${H}_{2}^{-}$ or ${H}_{2}^{+}$.

Our result is caused by the dynamic instability of the electronic subsystem. Note that the dynamic instability of the molecule is superimposed on the static instability for high values of *γ* parameter. We showed that the increase in the value of *γ* strongly reduces the dissociation energy of the molecule. Above ${\gamma }_{MS}=0.659374$ Ry, the molecule is in the metastable state, decaying definitively for ${\gamma }_{D} > 1.024638$ Ry.

An additional effect, that we observed for *γ* higher than ${\gamma }_{{\mathscr{P}}{\mathscr{T}}}=0.520873$ Ry, is the ${\mathscr{P}}{\mathscr{T}}$ symmetry breaking of the electronic Hamiltonian ${\hat{\mathscr{H}}}_{e\gamma }$. As a result, the two highest energies of the electron state assume complex values and the number of available electronic states of the molecule is reduced to four. This effect does not influence the stability of the considered system. Additionally, the ${\mathscr{P}}{\mathscr{T}}$ symmetry breaking does not change either the values of the integrals of the electronic Hamiltonian, or the phonon or rotational properties of the hydrogen molecules, or the electron-phonon interaction constants. The dynamics of the electronic subsystem is also independent on the breaking of the ${\mathscr{P}}{\mathscr{T}}$ symmetry of ${\hat{\mathscr{H}}}_{e\gamma }$.

It should be noted that all of the mentioned topics have more than just an academic value. Regarding the area of modern technology, particular attention should be paid to the nanoelectronic section linked to the molecular junctions^52–61^. Particularly interesting are the hydrogen molecule-bridged junctions of the type X/H_2_/X, where symbol X means metals like Pt^52,53,56–58^, Pd^55^, Au^58,59^, Cu^60^ or Ni^61^. It is obvious that in nanojunctions there is no issue with the stability of a molecular bridge interacting with environment, because of the whole system being stabilized by electrodes. However, it does not mean that the issue of reducing the molecular levels of the bridge caused by the correspondingly strong interaction of the hydrogen molecule with the electrodes of the joint can be omitted ($\gamma  > {\gamma }_{{\mathscr{P}}{\mathscr{T}}}^{^{\prime} }$, whereas ${\gamma }_{{\mathscr{P}}{\mathscr{T}}}^{^{\prime} }$ means the value of *γ* parameter for which the ${\mathscr{P}}{\mathscr T}$ symmetry breaking of the electronic bridge sub-system happens in the junction).

It should be emphasized that the formalism presented in the work enables the detailed analysis of the electronic structure of the hydrogen bridge in a nanojunction. For this purpose, the initial determination of physical parameters of the considered nanojunction, particularly of the equilibrium distance between the hydrogen atoms in the bridge (${R^{\prime} }_{0}$), should be done according to the method based on the density-functional theory (DFT)^62^. The physical state of the bridge when there is no flux (*γ* = 0) corresponds to the minimum enthalpy value: $H={E}_{p}+{E}_{e}+FR^{\prime}$, where the symbol *F* denotes the force exerted on the bridge by the junction anchors. The value of *F*, within the scheme presented in the work, should be selected so that the minimum enthalpy value is achieved for the ${R^{\prime} }_{0}$ distance. Further calculations in order to characterise the electronic structure of the bridge for *γ* ≠ 0 should be performed according to the presented scheme, applying the generalised formula for the enthalpy: $H={E}_{p}+{E}_{e\gamma }+FR^{\prime}$.

Noticeably, the dynamics of electronic observables of the molecular bridge interacting with electrodes should not be analyzed with the use of classical Heisenberg equation, but rather with a formalism of non-Hermitian quantum mechanics^16,25,51^.

### The eigenvalues of the electronic Hamiltonian of the hydrogen molecule interacting with the environment

The Hamiltonian ${\hat{\mathscr{H}}}_{e\gamma }$ should be written in the matrix form:

15 $$(\begin{array}{cccccc}{h}_{1}^{+} & 0 & t+V & 0 & J & t+V\\ 0 & {h}_{2} & 0 & 0 & 0 & 0\\ t+V & 0 & 2\varepsilon +K & 0 & t+V & -\,J\\ 0 & 0 & 0 & {h}_{2} & 0 & 0\\ J & 0 & t+V & 0 & {h}_{1}^{-} & t+V\\ t+V & 0 & -\,J & 0 & t+V & 2\varepsilon +K\end{array}).$$

where: ${h}_{1}^{\pm }=2\varepsilon +U\pm 2i\gamma$, and ${h}_{2}=2\varepsilon +K-J$. By using the operator (15) there was brought out the preliminary formulas for the eigenvalues, which has the form as follows:

16 $${E}_{1}=-\,J+K+2\varepsilon ,$$

17 $${E}_{2}=-\,J+K+2\varepsilon ,$$

18 $${E}_{3}=J+K+2\varepsilon ,$$

19 $$\begin{array}{rcl}{E}_{4} & = & \frac{1}{3}(\,-\,J+K+2U+[\,-\,4{J}^{2}+2J(K-U)\\  &  & -\,{(K-U)}^{2}+12(\,-\,{(t+V)}^{2}+{\gamma }^{2})]/[\,-\,8{J}^{3}+6{J}^{2}(K-U)\\  &  & +\,3J({(K-U)}^{2}+12(\,-{(t+V)}^{2}+{\gamma }^{2}))-(K-U)({(K-U)}^{2}\\  &  & +\,\mathrm{18((}t+V{)}^{2}+2{\gamma }^{2}))+\frac{1}{2}\sqrt{A-B}{]}^{\frac{1}{3}}\\  &  & -\,[\,-\,8{J}^{3}+6{J}^{2}(K-U)+3J((K-U{)}^{2}\\  &  & +\,\mathrm{12(}\,-\,{(t+V)}^{2}+{\gamma }^{2}))-(K-U)((K-U{)}^{2}\\  &  & +\,\mathrm{18((}t+V{)}^{2}+2{\gamma }^{2}))+\frac{1}{2}\sqrt{A-B}{]}^{\frac{1}{3}}+6\varepsilon ),\end{array}$$

20 $$\begin{array}{rcl}{E}_{5} & = & \frac{1}{12}\mathrm{([2(1}+i\sqrt{3}\mathrm{)(4}{J}^{2}+{(K-U)}^{2}+2J(\,-\,K+U)\\  &  & +\,\mathrm{12(}t+V-\gamma )(t+V+\gamma ))]/[\,-\,8{J}^{3}+6{J}^{2}(K-U)\\  &  & +\,3J((K-U{)}^{2}+\mathrm{12(}\,-\,{(t+V)}^{2}+{\gamma }^{2}))-(K-U)((K-U{)}^{2}\\  &  & +\,\mathrm{18((}t+V{)}^{2}+2{\gamma }^{2}))+\frac{1}{2}\sqrt{A-B}{]}^{\frac{1}{3}}+\mathrm{2(1}-i\sqrt{3})[\,-\,8{J}^{3}\\  &  & +\,6{J}^{2}(K-U)+3J((K-U{)}^{2}+12(\,-\,{(t+V)}^{2}+{\gamma }^{2}))\\  &  & -\,(K-U)((K-U{)}^{2}+\mathrm{18((}t+V{)}^{2}+2{\gamma }^{2}))+\frac{1}{2}\sqrt{A-B}{]}^{\frac{1}{3}}\\  &  & +\,\mathrm{4(}\,-\,J+K+2U+6\varepsilon )),\end{array}$$

21 $$\begin{array}{rcl}{E}_{6} & = & \frac{1}{12}\mathrm{([2(1}-i\sqrt{3}\mathrm{)(4}{J}^{2}+{(K-U)}^{2}+2J(\,-\,K+U)\\  &  & +\,\mathrm{12(}t+V-\gamma )(t+V+\gamma ))]/[-\,8{J}^{3}+6{J}^{2}(K-U)\\  &  & +\,3J((K-U{)}^{2}+\mathrm{12(}\,-\,{(t+V)}^{2}+{\gamma }^{2}))-(K-U)((K-U{)}^{2}\\  &  & +\,\mathrm{18((}t+V{)}^{2}+2{\gamma }^{2}))+\frac{1}{2}\sqrt{A-B}{]}^{\frac{1}{3}}+\mathrm{2(1}+i\sqrt{3})[\,-\,8{J}^{3}\\  &  & +\,6{J}^{2}(K-U)+3J((K-U{)}^{2}+\mathrm{12(}\,-\,{(t+V)}^{2}+{\gamma }^{2}))\\  &  & -\,(K-U)((K-U{)}^{2}+\mathrm{18((}t+V{)}^{2}+2{\gamma }^{2}))+\frac{1}{2}\sqrt{A-B}{]}^{\frac{1}{3}}\\  &  & +\,\mathrm{4(}\,-\,J+K+2U+6\varepsilon )),\end{array}$$

wherein:

22 $$A=4{[(2J+K-U)[(J-K+U)(4J-K+U)+18{(t+V)}^{2}]-36(J-K+U){\gamma }^{2}]}^{2},$$

and

23 $$B=4{[4{J}^{2}+{(K-U)}^{2}+2J(-K+U)+12(t+V-\gamma )(t+V+\gamma )]}^{3}.$$

**Figure 7:**
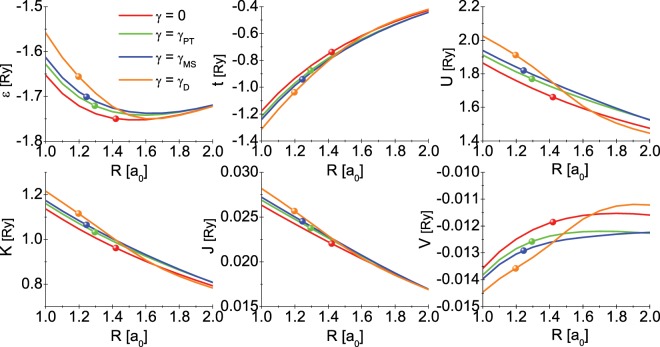
The integral of the Hamiltonian ${\hat{\mathscr{H}}}_{e\gamma }$ as a function of the inter-proton distance for the selected values of the parameter modeling the interaction of the molecule with the environment. The balls placed on the curves point to the equilibrium value of the inter-proton distance *R*_0_.

**Figure 8:**
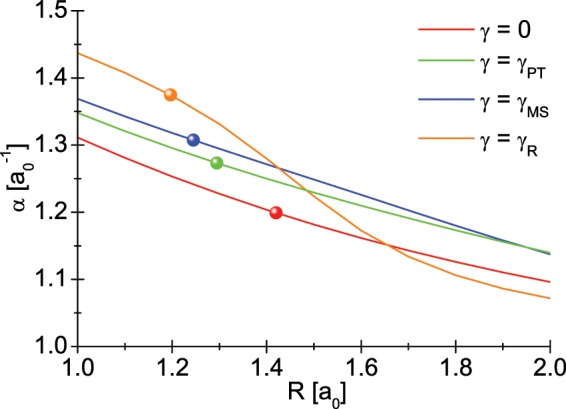
The variation parameter *α* as a function of the proton distance for selected *γ* values.

An attentive reader will notice that the energies *ε*, *t*, *U*, etc. are explicit functions of the inter-proton distance *R* and the parameter *α*. In the Fig. 7, we plotted the discussed values of the energies as the function of *R* and *γ*. Additionally, in Table 1 we give the equilibrium values of the ${\hat{\mathscr{H}}}_{e\gamma }$ parameters. The explicit dependence of the variational parameter *α* on the distance *R* is shown in the Fig. 8.

### The equilibrium values of phonon energy, rotational energy and the electron-phonon coupling function

In the Tables 2 and 3, we collected the equilibrium values of the phonon parameters for selected *γ*. The Table 4 presents the equilibrium values of the electron-phonon coupling functions. The Table 5 presents the equilibrium distance *R*_0_, the equilibrium inverse size of the orbital α_0_, and the ground-state energy *ET*(4)(*R*_*0*_) for different values of γ.

**Table 3:** The harmonic potential parameter *k*_*H*_ and the quantum energy for different values of *γ*.

*γ* [Ry]	*k*_*H*_ [${\bf{Ry}}/{{\boldsymbol{a}}}_{{\bf{0}}}^{{\bf{2}}}$]	${{\boldsymbol{\omega }}}_{{\bf{0}}}^{{\bf{H}}}$ [Ry]
**0**	**0.691719**	**0.027449**
0.1	0.379254	0.027886
0.2	0.387102	0.028463
0.3	0.402162	0.029570
0.4	0.412453	0.0303274
0.5	0.427453	0.0314304
${\gamma }_{{\mathscr{P}}{\mathscr{T}}}$ = **0.520873**	**0.919309**	**0.031644**
0.6	0.439769	0.0323359
${\gamma }_{{\mathcal M}{\mathcal S}}$ = **0.659374**	**0.980341**	**0.0326775**
0.7	0.0327805	0.445815
0.8	0.440037	0.0323556
0.9	0.409835	0.030135
1	0.29284	0.0215324
${\gamma }_{\mathscr D}$ = **1.024638**	**0.0050384**	**0.00234265**

**Table 4:** The values of the electron-ion coupling constants at the hydrogen-molecule equilibrium for different values of *γ*.

*γ* [Ry]	${g}_{{\varepsilon }_{0}}$ [Ry/a_0_]	${g}_{{t}_{0}}$ [Ry/a_0_]	${g}_{{U}_{0}}$ [Ry/a_0_]	${g}_{{K}_{0}}$ [Ry/a_0_]	${g}_{{J}_{0}}$ [Ry/a_0_]	${g}_{{V}_{0}}$ [Ry/a_0_]
**0**	**0.001744**	**0.609033**	**−0.126289**	**−0.236261**	**−0.007502**	**−0.000385**
0.1	**−**0.000858724	0.615157	**−**0.127093	**−**0.237581	**−**0.00752746	**−**0.000362566
0.2	**−**0.00860958	0.633159	**−**0.12944	**−**0.241435	**−**0.00760231	**−**0.000297894
0.3	**−**0.0213324	0.662009	**−**0.133151	**−**0.247532	**−**0.00772034	**−**0.000194141
0.4	**−**0.0387245	0.70024	**−**0.13798	**−**0.255475	**−**0.00787403	**−**0.0000572511
0.5	**−**0.0603282	0.746108	**−**0.143655	**−**0.264823	**−**0.00805519	0.000105421
${\gamma }_{{\mathscr{P}}{\mathscr{T}}}$ = **0.520873**	**−0.0653133**	**0.756473**	**−0.144921**	**−0.266911**	**−0.00809584**	**0.000141774**
0.6	**−**0.0854991	0.797725	**−**0.149904	**−**0.27515	**−**0.00825739	0.000284209
${\gamma }_{{\mathcal M}{\mathcal S}}$ = **0.659374**	**−0.101762**	**0.830226**	**−0.153775**	**−0.281576**	**−0.00838543**	**0.000392951**
0.7	**−**0.113288	0.852925	**−**0.156455	**−**0.286042	**−**0.00847612	0.000466248
0.8	**−**0.142132	0.908801	**−**0.162993	**−**0.297043	**−**0.00870995	0.00063131
0.9	**−**0.168739	0.95991	**−**0.168994	**−**0.307449	**−**0.00896312	0.000736959
1	**−**0.179745	0.984777	**−**0.17245	**−**0.314684	**−**0.0092668	0.000605461
${\gamma }_{\mathscr D}$ = **1.024638**	**−0.156139**	**0.947351**	**−0.169079**	**−0.311133**	**−0.00941164**	**0.000204999**

**Table 5:** The equilibrium distance *R*_0_, the equilibrium inverse size of the orbital *α*_0_, and the ground-state energy ${E}_{T}^{(4)}({R}_{0})$ for different values of *γ*.

*γ* [Ry]	*R*_0_ [a_0_]	*α*_0_ [${{\bf{a}}}_{{\bf{0}}}^{-{\bf{1}}}$]	${{\boldsymbol{E}}}_{{\boldsymbol{T}}}^{({\bf{4}})}$(*R*_0_) [Ry]
**0**	**1.41968**	**1.199206**	**−2.323011**
0.1	1.413598	1.202479	**−**2.314919
0.2	1.396223	1.211990	**−**2.290874
0.3	1.369845	1.22690	**−**2.251536
0.4	1.33742	1.24609	**−**2.19787
0.5	1.301859	1.268341	**−**2.131022
${\gamma }_{{\mathscr{P}}{\mathscr{T}}}$ = **0.520873**	**1.294281**	**1.273265**	**−2.115516**
0.6	1.265651	1.292526	**−**2.052185
*γ*_*MS*_ **= 0.659374**	**1.244701**	**1.307372**	**−2.000188**
0.7	1.230858	1.317603	**−**1.962537
0.8	1.199459	1.342514	**−**1.863195
0.9	1.174508	1.365733	**−**1.755232
1	1.168653	1.38188	**−**1.639820
*γ*_*D*_ = **1.024638**	**1.196587**	**1.374634**	**−1.610491**

### The set of the differential equations for electron observables (*γ* = 0)

The system of differential equations has the form:

24 $$\begin{array}{ccc}i\frac{d\langle {\hat{n}}_{1\uparrow }\rangle }{dT} & = & {t}_{1\uparrow }\langle {\hat{n}}_{12\uparrow }\rangle -{t}_{2\uparrow }\langle {\hat{n}}_{21\uparrow }\rangle +{J}_{1\uparrow }\langle {\hat{n}}_{1\uparrow \downarrow }\rangle \\  &  & -\,{J}_{1\downarrow }\langle {\hat{n}}_{1\downarrow \uparrow }\rangle +{P}_{1}\langle {\hat{\Delta }}_{1}^{\dagger }\rangle -{P}_{1}^{\ast }\langle {\hat{\Delta }}_{1}\rangle ,\end{array}$$

25 $$\begin{array}{ccc}i\frac{d\langle {\hat{n}}_{1\downarrow }\rangle }{dT} & = & {t}_{1\downarrow }\langle {\hat{n}}_{12\downarrow }\rangle -{t}_{2\downarrow }\langle {\hat{n}}_{21\downarrow }\rangle +{J}_{1\downarrow }\langle {\hat{n}}_{1\downarrow \uparrow }\rangle \\  &  & -\,{J}_{1\uparrow }\langle {\hat{n}}_{1\uparrow \downarrow }\rangle +{P}_{1}\langle {\hat{\Delta }}_{1}^{\dagger }\rangle -{P}_{1}^{\ast }\langle {\hat{\Delta }}_{1}\rangle ,\end{array}$$

26 $$\begin{array}{rcl}i\frac{d\langle {\hat{n}}_{2\uparrow }\rangle }{dT} & = & -{t}_{1\uparrow }\langle {\hat{n}}_{12\uparrow }\rangle +{t}_{2\uparrow }\langle {\hat{n}}_{21\uparrow }\rangle +{J}_{2\uparrow }\langle {\hat{n}}_{2\uparrow \downarrow }\rangle -{J}_{2\downarrow }\langle {\hat{n}}_{2\downarrow \uparrow }\rangle \\  &  & +\,{P}_{2}\langle {\hat{\Delta }}_{2}^{\dagger }\rangle -{P}_{2}^{\ast }\langle {\hat{\Delta }}_{2}\rangle ,\end{array}$$

27 $$\begin{array}{rcl}i\frac{d\langle {\hat{n}}_{2\downarrow }\rangle }{dT} & = & -{t}_{1\downarrow }\langle {\hat{n}}_{12\downarrow }\rangle +{t}_{2\downarrow }\langle {\hat{n}}_{21\downarrow }\rangle +{J}_{2\downarrow }\langle {\hat{n}}_{2\downarrow \uparrow }\rangle \\  &  & -\,{J}_{2\uparrow }\langle {\hat{n}}_{2\uparrow \downarrow }\rangle +{P}_{2}\langle {\hat{\Delta }}_{2}^{\dagger }\rangle -{P}_{2}^{\ast }\langle {\hat{\Delta }}_{2}\rangle ,\end{array}$$

28 $$i\frac{d\langle {\hat{n}}_{12\uparrow }\rangle }{dT}=-\,{\varepsilon }_{1\uparrow }\langle {\hat{n}}_{12\uparrow }\rangle +{\varepsilon }_{2\uparrow }\langle {\hat{n}}_{12\uparrow }\rangle +{t}_{2\uparrow }\langle {\hat{n}}_{1\uparrow }\rangle -{t}_{2\uparrow }\langle {\hat{n}}_{2\uparrow }\rangle ,$$

29 $$i\frac{d\langle {\hat{n}}_{12\downarrow }\rangle }{dT}=-\,{\varepsilon }_{1\downarrow }\langle {\hat{n}}_{12\downarrow }\rangle +{\varepsilon }_{2\downarrow }\langle {\hat{n}}_{12\downarrow }\rangle +{t}_{2\downarrow }\langle {\hat{n}}_{1\downarrow }\rangle -{t}_{2\downarrow }\langle {\hat{n}}_{2\downarrow }\rangle ,$$

30 $$i\frac{d\langle {\hat{n}}_{21\uparrow }\rangle }{dT}={\varepsilon }_{1\uparrow }\langle {\hat{n}}_{21\uparrow }\rangle -{\varepsilon }_{2\uparrow }\langle {\hat{n}}_{21\uparrow }\rangle +{t}_{1\uparrow }\langle {\hat{n}}_{2\uparrow }\rangle -{t}_{1\uparrow }\langle {\hat{n}}_{1\uparrow }\rangle ,$$

31 $$i\frac{d\langle {\hat{n}}_{21\downarrow }\rangle }{dT}={\varepsilon }_{1\downarrow }\langle {\hat{n}}_{21\downarrow }\rangle -{\varepsilon }_{2\downarrow }\langle {\hat{n}}_{21\downarrow }\rangle +{t}_{1\downarrow }\langle {\hat{n}}_{2\downarrow }\rangle -{t}_{1\downarrow }\langle {\hat{n}}_{1\downarrow }\rangle ,$$

32 $$i\frac{d\langle {\hat{n}}_{1\uparrow \downarrow }\rangle }{dT}=-\,{\varepsilon }_{1\uparrow }\langle {\hat{n}}_{1\uparrow \downarrow }\rangle +{\varepsilon }_{1\downarrow }\langle {\hat{n}}_{1\uparrow \downarrow }\rangle +{J}_{1\downarrow }\langle {\hat{n}}_{1\uparrow }\rangle -{J}_{1\downarrow }\langle {\hat{n}}_{1\downarrow }\rangle ,$$

33 $$i\frac{d\langle {\hat{n}}_{1\downarrow \uparrow }\rangle }{dT}={\varepsilon }_{1\uparrow }\langle {\hat{n}}_{1\downarrow \uparrow }\rangle -{\varepsilon }_{1\downarrow }\langle {\hat{n}}_{1\downarrow \uparrow }\rangle +{J}_{1\uparrow }\langle {\hat{n}}_{1\downarrow }\rangle -{J}_{1\uparrow }\langle {\hat{n}}_{1\uparrow }\rangle ,$$

34 $$i\frac{d\langle {\hat{n}}_{2\uparrow \downarrow }\rangle }{dT}=-\,{\varepsilon }_{2\uparrow }\langle {\hat{n}}_{2\uparrow \downarrow }\rangle +{\varepsilon }_{2\downarrow }\langle {\hat{n}}_{2\uparrow \downarrow }\rangle +{J}_{2\downarrow }\langle {\hat{n}}_{2\uparrow }\rangle -{J}_{2\downarrow }\langle {\hat{n}}_{2\downarrow }\rangle ,$$

35 $$i\frac{d\langle {\hat{n}}_{2\downarrow \uparrow }\rangle }{dT}={\varepsilon }_{2\uparrow }\langle {\hat{n}}_{2\downarrow \uparrow }\rangle -{\varepsilon }_{2\downarrow }\langle {\hat{n}}_{2\downarrow \uparrow }\rangle +{J}_{2\uparrow }\langle {\hat{n}}_{2\downarrow }\rangle -{J}_{2\uparrow }\langle {\hat{n}}_{2\uparrow }\rangle ,$$

36 $$i\frac{d\langle {\hat{\Delta }}_{1}^{\dagger }\rangle }{dT}=-\,{\varepsilon }_{1\uparrow }\langle {\hat{\Delta }}_{1}^{\dagger }\rangle -{\varepsilon }_{1\downarrow }\langle {\hat{\Delta }}_{1}^{\dagger }\rangle +{P}_{1}^{\ast }\langle {\hat{n}}_{1\uparrow }\rangle +{P}_{1}^{\ast }\langle {\hat{n}}_{1\downarrow }\rangle -{P}_{1}^{\ast },$$

37 $$i\frac{d\langle {\hat{\Delta }}_{1}\rangle }{dT}={\varepsilon }_{1\uparrow }\langle {\hat{\Delta }}_{1}\rangle +{\varepsilon }_{1\downarrow }\langle {\hat{\Delta }}_{1}\rangle -{P}_{1}\langle {\hat{n}}_{1\downarrow }\rangle -{P}_{1}\langle {\hat{n}}_{1\uparrow }\rangle +{P}_{1},$$

38 $$i\frac{d\langle {\hat{\Delta }}_{2}^{\dagger }\rangle }{dT}=-\,{\varepsilon }_{2\uparrow }\langle {\hat{\Delta }}_{2}^{\dagger }\rangle -{\varepsilon }_{2\downarrow }\langle {\hat{\Delta }}_{2}^{\dagger }\rangle +{P}_{2}^{\ast }\langle {\hat{n}}_{2\uparrow }\rangle +{P}_{2}^{\ast }\langle {\hat{n}}_{2\downarrow }\rangle -{P}_{2}^{\ast },$$

39 $$i\frac{d\langle {\hat{\Delta }}_{2}\rangle }{dT}={\varepsilon }_{2\uparrow }\langle {\hat{\Delta }}_{2}\rangle +{\varepsilon }_{2\downarrow }\langle {\hat{\Delta }}_{2}\rangle -{P}_{2}\langle {\hat{n}}_{2\downarrow }\rangle -{P}_{2}\langle {\hat{n}}_{2\uparrow }\rangle +{P}_{2}.$$

### The system of differential equations for electron observables (*γ* ≠ 0)

The system of differential equations can be written in the form:

40 $$\begin{array}{rcl}i\frac{d\langle {\hat{n}}_{1\uparrow }\rangle }{dT} & = & {t}_{1\uparrow }\langle {\hat{n}}_{12\uparrow }\rangle -{t}_{2\uparrow }\langle {\hat{n}}_{21\uparrow }\rangle +{J}_{1\uparrow }\langle {\hat{n}}_{1\uparrow \downarrow }\rangle -{J}_{1\downarrow }\langle {\hat{n}}_{1\downarrow \uparrow }\rangle \\  &  & +\,{P}_{1}\langle {\hat{\Delta }}_{1}^{\dagger }\rangle -{P}_{1}^{\ast }\langle {\hat{\Delta }}_{1}\rangle +i\gamma \langle {\hat{n}}_{1\uparrow }\rangle -2i\gamma |{\Delta }_{1}{|}^{2}\\  &  & -\,2i\gamma  < {\hat{n}}_{1\uparrow } > \sum _{\sigma }(\langle {\hat{n}}_{1\sigma }\rangle -\langle {\hat{n}}_{2\sigma }\rangle ),\end{array}$$

41 $$\begin{array}{rcl}i\frac{d\langle {\hat{n}}_{1\downarrow }\rangle }{dT} & = & {t}_{1\downarrow }\langle {\hat{n}}_{12\downarrow }\rangle -{t}_{2\downarrow }\langle {\hat{n}}_{21\downarrow }\rangle +{J}_{1\downarrow }\langle {\hat{n}}_{1\downarrow \uparrow }\rangle -{J}_{1\uparrow }\langle {\hat{n}}_{1\uparrow \downarrow }\rangle \\  &  & +\,i\gamma \langle {\hat{n}}_{1\downarrow }\rangle -2i\gamma |{\Delta }_{1}{|}^{2}+{P}_{1}\langle {\hat{\Delta }}_{1}^{\dagger }\rangle -{P}_{1}^{\ast }\langle {\hat{\Delta }}_{1}\rangle \\  &  & -\,2i\gamma \langle {\hat{n}}_{1\downarrow }\rangle \sum _{\sigma }\,(\langle {\hat{n}}_{1\sigma }\rangle -\langle {\hat{n}}_{2\sigma }\rangle ),\end{array}$$

42 $$\begin{array}{rcl}i\frac{d\langle {\hat{n}}_{2\uparrow }\rangle }{dT} & = & -{t}_{1\uparrow }\langle {\hat{n}}_{12\uparrow }\rangle +{t}_{2\uparrow }\langle {\hat{n}}_{21\uparrow }\rangle +{J}_{2\uparrow }\langle {\hat{n}}_{2\uparrow \downarrow }\rangle -{J}_{2\downarrow }\langle {\hat{n}}_{2\downarrow \uparrow }\rangle \\  &  & +\,{P}_{2}\langle {\hat{\Delta }}_{2}^{\dagger }\rangle -{P}_{2}^{\ast }\langle {\hat{\Delta }}_{2}\rangle -i\gamma \langle {\hat{n}}_{2\uparrow }\rangle +2i\gamma |{\Delta }_{2}{|}^{2}\\  &  & -\,2i\gamma \langle {\hat{n}}_{2\uparrow }\rangle \sum _{\sigma }\,(\langle {\hat{n}}_{1\sigma }\rangle -\langle {\hat{n}}_{2\sigma }\rangle ),\end{array}$$

43 $$\begin{array}{rcl}i\frac{d\langle {\hat{n}}_{2\downarrow }\rangle }{dT} & = & -{t}_{1\downarrow }\langle {\hat{n}}_{12\downarrow }\rangle +{t}_{2\downarrow }\langle {\hat{n}}_{21\downarrow }\rangle +{J}_{2\downarrow }\langle {\hat{n}}_{2\downarrow \uparrow }\rangle -{J}_{2\uparrow }\langle {\hat{n}}_{2\uparrow \downarrow }\rangle \\  &  & +\,{P}_{2}\langle {\hat{\Delta }}_{2}^{\dagger }\rangle -{P}_{2}^{\ast }\langle {\hat{\Delta }}_{2}\rangle -i\gamma \langle {\hat{n}}_{2\downarrow }\rangle +2i\gamma |{\Delta }_{2}{|}^{2}\\  &  & -\,2i\gamma \langle {\hat{n}}_{2\downarrow }\rangle \sum _{\sigma }\,(\langle {\hat{n}}_{1\sigma }\rangle -\langle {\hat{n}}_{2\sigma }\rangle ),\end{array}$$

44 $$\begin{array}{rcl}i\frac{d\langle {\hat{n}}_{12\uparrow }\rangle }{dT} & = & -{\varepsilon }_{1\uparrow }\langle {\hat{n}}_{12\uparrow }\rangle +{\varepsilon }_{2\uparrow }\langle {\hat{n}}_{12\uparrow }\rangle +{t}_{2\uparrow }\langle {\hat{n}}_{1\uparrow }\rangle -{t}_{2\uparrow }\langle {\hat{n}}_{2\uparrow }\rangle \\  &  & +\,i\gamma \langle {\hat{n}}_{12\uparrow }\rangle \\  &  & -\,2i\gamma \langle {\hat{n}}_{12\uparrow }\rangle \sum _{\sigma }\,(\langle {\hat{n}}_{1\sigma }\rangle -\langle {\hat{n}}_{2\sigma }\rangle ),\end{array}$$

45 $$\begin{array}{rcl}i\frac{d\langle {\hat{n}}_{12\downarrow }\rangle }{dT} & = & -{\varepsilon }_{1\downarrow }\langle {\hat{n}}_{12\downarrow }\rangle +{\varepsilon }_{2\downarrow }\langle {\hat{n}}_{12\downarrow }\rangle +{t}_{2\downarrow }\langle {\hat{n}}_{1\downarrow }\rangle -{t}_{2\downarrow }\langle {\hat{n}}_{2\downarrow }\rangle \\  &  & +\,i\gamma \langle {\hat{n}}_{12\downarrow }\rangle \\  &  & -\,2i\gamma \langle {\hat{n}}_{12\downarrow }\rangle \sum _{\sigma }\,(\langle {\hat{n}}_{1\sigma }\rangle -\langle {\hat{n}}_{2\sigma }\rangle ),\end{array}$$

46 $$\begin{array}{rcl}i\frac{d\langle {\hat{n}}_{21\uparrow }\rangle }{dT} & = & {\varepsilon }_{1\uparrow }\langle {\hat{n}}_{21\uparrow }\rangle -{\varepsilon }_{2\uparrow }\langle {\hat{n}}_{21\uparrow }\rangle +{t}_{1\uparrow }\langle {\hat{n}}_{2\uparrow }\rangle -{t}_{1\uparrow }\langle {\hat{n}}_{1\uparrow }\rangle \\  &  & -\,i\gamma \langle {\hat{n}}_{21\uparrow }\rangle \\  &  & -\,2i\gamma \langle {\hat{n}}_{21\uparrow }\rangle \sum _{\sigma }\,(\langle {\hat{n}}_{1\sigma }\rangle -\langle {\hat{n}}_{2\sigma }\rangle ),\end{array}$$

47 $$\begin{array}{rcl}i\frac{d\langle {\hat{n}}_{21\downarrow }\rangle }{dT} & = & {\varepsilon }_{1\downarrow }\langle {\hat{n}}_{21\downarrow }\rangle -{\varepsilon }_{2\downarrow }\langle {\hat{n}}_{21\downarrow }\rangle +{t}_{1\downarrow }\langle {\hat{n}}_{2\downarrow }\rangle -{t}_{1\downarrow }\langle {\hat{n}}_{1\downarrow }\rangle \\  &  & -\,i\gamma \langle {\hat{n}}_{21\downarrow }\rangle \\  &  & -\,2i\gamma \langle {\hat{n}}_{21\downarrow }\rangle \sum _{\sigma }\,(\langle {\hat{n}}_{1\sigma }\rangle -\langle {\hat{n}}_{2\sigma }\rangle ),\end{array}$$

48 $$\begin{array}{rcl}i\frac{d\langle {\hat{n}}_{1\uparrow \downarrow }\rangle }{dT} & = & -{\varepsilon }_{1\uparrow }\langle {\hat{n}}_{1\uparrow \downarrow }\rangle +{\varepsilon }_{1\downarrow }\langle {\hat{n}}_{1\uparrow \downarrow }\rangle +{J}_{1\downarrow }\langle {\hat{n}}_{1\uparrow }\rangle -{J}_{1\downarrow }\langle {\hat{n}}_{1\downarrow }\rangle \\  &  & +\,i\gamma \langle {\hat{n}}_{1\uparrow \downarrow }\rangle \\  &  & -\,2i\gamma \langle {\hat{n}}_{1\uparrow \downarrow }\rangle \sum _{\sigma }\,(\langle {\hat{n}}_{1\sigma }\rangle -\langle {\hat{n}}_{2\sigma }\rangle ),\end{array}$$

49 $$\begin{array}{rcl}i\frac{d\langle {\hat{n}}_{1\downarrow \uparrow }\rangle }{dT} & = & {\varepsilon }_{1\uparrow }\langle {\hat{n}}_{1\downarrow \uparrow }\rangle -{\varepsilon }_{1\downarrow }\langle {\hat{n}}_{1\downarrow \uparrow }\rangle +{J}_{1\uparrow }\langle {\hat{n}}_{1\downarrow }\rangle -{J}_{1\uparrow }\langle {\hat{n}}_{1\uparrow }\rangle \\  &  & +\,i\gamma \langle {\hat{n}}_{1\downarrow \uparrow }\rangle \\  &  & -\,2i\gamma \langle {\hat{n}}_{1\downarrow \uparrow }\rangle \sum _{\sigma }\,(\langle {\hat{n}}_{1\sigma }\rangle -\langle {\hat{n}}_{2\sigma }\rangle ),\end{array}$$

50 $$\begin{array}{rcl}i\frac{d\langle {\hat{n}}_{2\uparrow \downarrow }\rangle }{dT} & = & -{\varepsilon }_{2\uparrow }\langle {\hat{n}}_{2\uparrow \downarrow }\rangle +{\varepsilon }_{2\downarrow }\langle {\hat{n}}_{2\uparrow \downarrow }\rangle +{J}_{2\downarrow }\langle {\hat{n}}_{2\uparrow }\rangle -{J}_{2\downarrow }\langle {\hat{n}}_{2\downarrow }\rangle \\  &  & -\,i\gamma \langle {\hat{n}}_{2\uparrow \downarrow }\rangle \\  &  & -\,2i\gamma \langle {\hat{n}}_{2\uparrow \downarrow }\rangle \sum _{\sigma }\,(\langle {\hat{n}}_{1\sigma }\rangle -\langle {\hat{n}}_{2\sigma }\rangle ),\end{array}$$

51 $$\begin{array}{rcl}i\frac{d\langle {\hat{n}}_{2\downarrow \uparrow }\rangle }{dT} & = & {\varepsilon }_{2\uparrow }\langle {\hat{n}}_{2\downarrow \uparrow }\rangle -{\varepsilon }_{2\downarrow }\langle {\hat{n}}_{2\downarrow \uparrow }\rangle +{J}_{2\uparrow }\langle {\hat{n}}_{2\downarrow }\rangle -{J}_{2\uparrow }\langle {\hat{n}}_{2\uparrow }\rangle \\  &  & -\,i\gamma \langle {\hat{n}}_{2\downarrow \uparrow }\rangle \\  &  & -\,2i\gamma \langle {\hat{n}}_{2\downarrow \uparrow }\rangle \sum _{\sigma }\,(\langle {\hat{n}}_{1\sigma }\rangle -\langle {\hat{n}}_{2\sigma }\rangle ),\end{array}$$

52 $$\begin{array}{rcl}i\frac{d\langle {\hat{\Delta }}_{1}^{\dagger }\rangle }{dT} & = & -{\varepsilon }_{1\uparrow }\langle {\hat{\Delta }}_{1}^{\dagger }\rangle -{\varepsilon }_{1\downarrow }\langle {\hat{\Delta }}_{1}^{\dagger }\rangle +{P}_{1}^{\ast }\langle {\hat{n}}_{1\uparrow }\rangle +{P}_{1}^{\ast }\langle {\hat{n}}_{1\downarrow }\rangle -{P}_{1}^{\ast }\\  &  & +\,2i\gamma \langle {\hat{\Delta }}_{1}^{\dagger }\rangle +2i\gamma \langle {\hat{\Delta }}_{1}^{\dagger }\rangle \langle {\hat{n}}_{1\downarrow }\rangle \\  &  & -\,2i\gamma \langle {\hat{\Delta }}_{1}^{\dagger }\rangle \sum _{\sigma }\,(\langle {\hat{n}}_{1\sigma }\rangle -\langle {\hat{n}}_{2\sigma }\rangle ),\end{array}$$

53 $$\begin{array}{rcl}i\frac{d\langle {\hat{\Delta }}_{1}\rangle }{dT} & = & {\varepsilon }_{1\uparrow }\langle {\hat{\Delta }}_{1}\rangle +{\varepsilon }_{1\downarrow }\langle {\hat{\Delta }}_{1}\rangle -{P}_{1}\langle {\hat{n}}_{1\downarrow }\rangle -{P}_{1}\langle {\hat{n}}_{1\uparrow }\rangle +{P}_{1}\\  &  & -\,i\gamma \langle {\hat{\Delta }}_{1}\rangle +2i\gamma \langle {\hat{\Delta }}_{1}\rangle \langle {\hat{n}}_{1\downarrow }\rangle \\  &  & -\,2i\gamma \langle {\hat{\Delta }}_{1}\rangle \sum _{\sigma }\,(\langle {\hat{n}}_{1\sigma }\rangle -\langle {\hat{n}}_{2\sigma }\rangle ),\end{array}$$

54 $$\begin{array}{rcl}i\frac{d\langle {\hat{\Delta }}_{2}^{\dagger }\rangle }{dT} & = & -{\varepsilon }_{2\uparrow }\langle {\hat{\Delta }}_{2}^{\dagger }\rangle -{\varepsilon }_{2\downarrow }\langle {\hat{\Delta }}_{2}^{\dagger }\rangle +{P}_{2}^{\ast }\langle {\hat{n}}_{2\uparrow }\rangle +{P}_{2}^{\ast }\langle {\hat{n}}_{2\downarrow }\rangle -{P}_{2}^{\ast }\\  &  & -\,2i\gamma \langle {\hat{\Delta }}_{2}^{\dagger }\rangle -2i\gamma \langle {\hat{\Delta }}_{2}^{\dagger }\rangle \langle {\hat{n}}_{2\downarrow }\rangle \\  &  & -\,2i\gamma \langle {\hat{\Delta }}_{2}^{\dagger }\rangle \sum _{\sigma }\,(\langle {\hat{n}}_{1\sigma }\rangle -\langle {\hat{n}}_{2\sigma }\rangle ),\end{array}$$

55 $$\begin{array}{rcl}i\frac{d\langle {\hat{\Delta }}_{2}\rangle }{dT} & = & {\varepsilon }_{2\uparrow }\langle {\hat{\Delta }}_{2}\rangle +{\varepsilon }_{2\downarrow }\langle {\hat{\Delta }}_{2}\rangle -{P}_{2}\langle {\hat{n}}_{2\downarrow }\rangle -{P}_{2}\langle {\hat{n}}_{2\uparrow }\rangle +{P}_{2}\\  &  & +\,i\gamma \langle {\hat{\Delta }}_{2}\rangle -2i\gamma \langle {\hat{\Delta }}_{2}\rangle \langle {\hat{n}}_{2\downarrow }\rangle \\  &  & -\,2i\gamma \langle {\hat{\Delta }}_{2}\rangle \sum _{\sigma }\,(\langle {\hat{n}}_{1\sigma }\rangle -\langle {\hat{n}}_{2\sigma }\rangle )\mathrm{}.\end{array}$$
