# Publisher Correction: Interaction of the hydrogen molecule with the environment: stability of the system and the ${\mathscr{P}}{\mathscr{T}}$ symmetry breaking

**DOI:** 10.1038/s41598-020-61865-8

**Published:** 2020-03-19

**Authors:** I. A. Wrona, M. W. Jarosik, R. Szczȩśniak, K. A. Szewczyk, M. K. Stala, W. Leoński

**Affiliations:** 1 Institute of Physics, Jan Długosz University in Czȩstochowa, Ave. Armii Krajowej 13/15, 42-200 Czȩstochowa, Poland; 2 Institute of Physics, Czȩstochowa University of Technology, Ave. Armii Krajowej 19, 42-200 Czȩstochowa, Poland; 3 Quantum Optics and Engineering Division, Faculty of Physics and Astronomy, University of Zielona Góra, Prof. Z. Szafrana 4a, 65-516 Zielona Góra, Poland; 4 Joint Laboratory of Optics of Palacký University and Institute of Physics of CAS, RCPTM, Faculty of Science, Palacký University, 17. listopadu 12, 771 46 Olomouc, Czech Republic

Correction to: *Scientific Reports* 10.1038/s41598-019-56849-2, published online 14 January 2020

The original version of this Article contained an error in Affiliation 2, which was incorrectly given as ‘Institute of Physics, Jan Długosz University, Ave. Armii Krajowej 19, 42-200, Czȩstochowa, Poland’. The correct affiliation is listed below:

Institute of Physics, Czȩstochowa University of Technology, Ave. Armii Krajowej 19, 42-200 Czȩstochowa, Poland.

This error has now been corrected in the HTML and PDF versions of the Article.

